# Re-Rising of Total Bilirubin Level after Postoperative Day 3 (The V Pattern) Predicting Liver Failure and Survival of Patients who Underwent Hepatectomy for Cholangiocarcinoma

**DOI:** 10.31557/APJCP.2020.21.12.3573

**Published:** 2020-12

**Authors:** Weerin Sawangkajohn, Vor Luvira, Natwutpong Leeratanakachorn, Theerawee Tipwaratorn, Suapa Theerakul, Apiwat Jarearnrat, Attapol Titapun, Tharatip Srisuk, Ake Pugkhem, Narong Khuntikeo, Vajarabhongsa Bhudhisawasdi, Supot Kamsa-Ard

**Affiliations:** 1 *Department of Surgery, Faculty of Medicine, Khon Kaen University, Khon Kaen, Thailand. *; 2 *Department of Surgery, Saraburi Hospital, Saraburi, Thailand. *; 3 *Department of Epidemiology and Biostatistics, Faculty of Public Health, Khon Kaen University, Khon Kaen, Thailand. *

**Keywords:** Cholangiocarcinoma, liver failure, bilirubin, survival, hepatectomy

## Abstract

**Objective:**

All types of cholangiocarcinoma (CCA) require a major hepatectomy, which has many post-operative complications. All complications usually present with persistent hyperbilirubinemia; however, studies on the prediction of post-operative hyperbilirubinemia after hepatectomy for patients with CCA are lacking. We evaluated the causes and patterns of persistent hyperbilirubinemia among the patients who underwent hepatectomy for CCA.

**Methods:**

We retrospectively reviewed the records of 216 CCA patients who underwent curative-intent hepatic resection between January 2015 and December 2016. We identified five patterns of hyperbilirubinemia for predicting the cause of persistent hyperbilirubinemia and the respective patient outcome. All clinical parameters and outcomes were analyzed for any significant associations.

**Results:**

Twenty-eight patients (24%) had post-operative persistent hyperbilirubinemia. Of these, liver failure was the most common cause (42.9%), followed by bile leakage (14.3%), then cholangitis (3.6%). Re-rising of the bilirubin level after post-operative day 3(the ‘V’ pattern), very well predicted liver failure. Moreover, this pattern was associated with poor survival of the patient.

**Conclusion:**

The current study provided a picture of persistent hyperbilirubinemia after hepatectomy for CCA. The proportion of post-operative liver failure was 12 percent. The pattern of serum bilirubin level could be used as a predictor of liver failure and long-term outcomes of CCA patients. The ‘V’ pattern was significantly associated with a high rate of liver failure and poor survival.

## Introduction

The mainstay of treatment for patients with all types of cholangiocarcinoma is major hepatectomy (Bridgewater et al., 2014; Titapun et al., 2015; Luvira et al., 2016; Luvira et al., 2017), which commonly leads to several post-operative complications (Hirashita et al., 2013; Dumitrascu et al., 2016). Common post-operative complications-which include post-operative liver failure, bile leakage, biliary stricture and acute cholangitis-usually present with hyperbilirubinemia (Liu et al., 2017). Since each complication requires different treatment (Russel, 2015; Huang et al., 2019), every attempt should be made for a precise diagnosis of the complication (s). 

There have been many attempts to determine the predictors of post-operative liver failure, using pre- and intra-operative parameters, including number of blood transfusions (Dumitrascu et al., 2016), intra-operative blood loss, operation time (Fukumori et al., 2011), platelet count, and calculated future liver remnant (Hirashita et al., 2013). None of the above represent any post-operative parameters for the prediction, especially for the patients with cholangiocarcinoma (CCA), who require more extensive liver resection and have some specific, severe, morbid complications. Studies on the prediction of post-operative hyperbilirubinemia after hepatectomy for the patient with CCA are lacking. 

To achieve a better understanding of the persistent hyperbilirubinemia after hepatectomy for CCA, we investigated the causes and nature of post-operative persistent hyperbilirubinemia. In particular, we evaluated the pattern of persistent hyperbilirubinemia in order to predict the causes of hyperbilirubinemia and the outcome of affected patients. 

## Materials and Methods

This retrospective study ran between January 2015 and December 2016. We retrospectively reviewed prospectively collected medical records of all consecutive histologic-confirmed CCA patients who underwent hepatectomy at Srinagarind Hospital, Faculty of Medicine, Khon Kaen University. We reviewed the clinical and laboratory data of 116 consecutive patients with respect to age, sex, pre- and postoperative liver function, tumor markers, complications, and survival.

*Our protocol of treatment of cholangiocarcinoma*

Prior to surgery, all patients were evaluated for resectability of the tumor and the type of resection, using cross-sectional imaging (i.e., computed tomography, magnetic resonance imaging, or both). All patients with obstructive jaundice received preoperative biliary drainage, either percutaneously or endoscopically, before surgery with aimed to reduce the total bilirubin level to below 10 mg/dl. All surgical procedures aimed to achieve gross tumor removal, and thus major hepatectomy was the first choice. All surgical specimens were sent to the Department of Pathology for pathological diagnosis and staging.

*Post-operative care.*

All patients were routinely admitted to the Intensive Care Unit at least 1 day post-operatively. The laboratory tests including liver function test, complete blood count, blood urea nitrogen, and creatinine were routinely performed at post-operative day (POD) 1, 3, 5, and 7. Enteral nutritional was initiated as soon as the patient could tolerate it. All drainage tubes were removed when no longer indicated. We supplemented branch chain amino acid to the patients diagnosed with post-operative liver failure.

*The definition of post-operative complications*

We defined the post-operative liver failure as any abnormal of liver synthetic function, which included serum total bilirubin more than 3 mg/dl and INR > 1.5, at POD5 without any documented cause (Rahbari et al., 2011). Post-operative bile leakage was defined as the drain- serum bilirubin ratio of > 3 at POD3 or the collection of bilious intra-abdominal fluid. We defined post-operative stricture when the stricture was confirmed by radiological evidence. Acute cholangitis was diagnosed according to the definition of the 2018 Tokyo Guideline for diagnosis of acute cholangitis (Kiriyama et al., 2018).

*Patterns of total bilirubin level*

**Figure 1:**
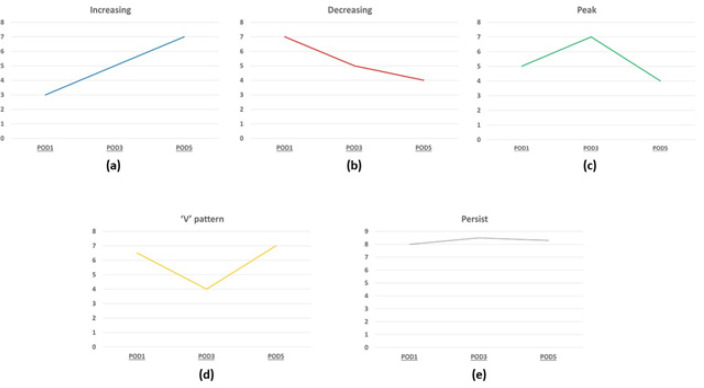
Patterns of Persistent Hyperbilirubinemia. a) increasing b) decreasing c) ‘peak’ pattern d) ‘V’ pattern e) persistent

**Figure 2:**
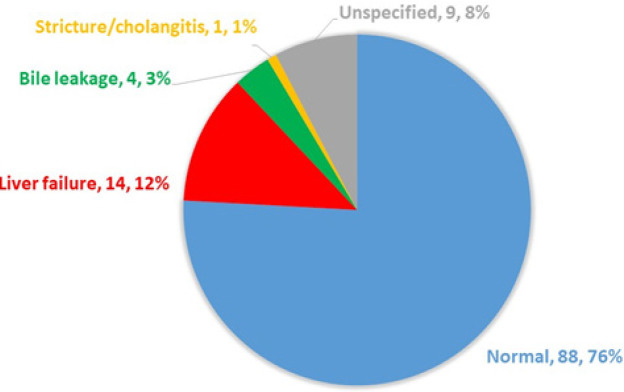
Proportion of Persistent Hyperbilirubinemia and Its Causes

**Figure3:**
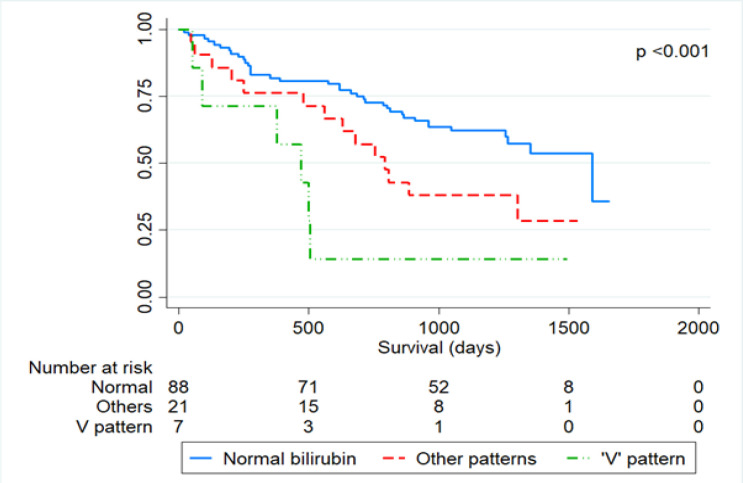
Kaplan-Meier Survival Curve of Cholangiocarcinoma Patients Treated by Hepatic Resection Stratified by the Pattern of Persistent Hyperbilirubinemia

The definition of persistent hyperbilirubinemia was a serum total bilirubin level > 3 mg/dl at the 5^th^ post-operative day. We classified the pattern of persistent hyperbilirubinemia into 5 groups according to the increasing serum bilirubin level at POD1, POD3, and POD5: 1) Increasing – continuously increasing from POD1 to POD5 (Figure 1a); 2) Decreasing – continuously decreasing from POD1 to POD5 (Figure 1b); 3) Peak – increasing to a peak at POD3 then decreasing at POD5 (Figure 1c); 4) ‘V’ – dropping to a nadir at POD3 then rising again at POD5 (Figure 1d); 5) Persistent – an abnormal level with no significant change > 0.5 mg/dl between POD1 and POD5 (Figure 1e).

*Ethical considerations*

The Institutional Review Board (IRB), Office of Human Research Ethics, Khon Kaen University, reviewed and approved the present study (HE611243).

## Results

*Clinical, demographic and operative data*

Of the 116 patients who underwent hepatic resection for cholangiocarcinoma (CCA), most were male (n=72. 62.1%) and the mean age was 62.03 (±8.25) years. Perihilar CCA outnumbered intrahepatic CCA (55.2 vs. 44.8%). The most common tumor morphology was intraductal (61.2%). Most of the hepatic resections were performed to the right side (66.3%) and right hepatectomy was the most common procedure (40.5%). Vascular resection was performed in 8 cases (6.9%). Less than half of the patients required vascular inflow occlusion during the operation (39.7%). The mean post-operative hospital stay was 13.1 (±7.4) days.

Eighty-eight (76%) of the patients had a normal serum total bilirubin level at POD5. Of the 28 patients with persistent hyperbilirubinemia, liver failure was the most common cause. The respective number of patients with liver failure, bile leakage, and cholangitis was 14 (12%), 4 (3%), and 1 (1%) (Figure 2). Nine patients (8%) had an unspecified cause of persistent hyperbilirubinemia despite having undergone complete investigations, and the level of serum bilirubin decreased overtime to a normal level without any clinically significant complications. Most of the patients who suffered from persistent hyperbilirubinemia were those who underwent hepatic resection for perihilar CCA, and only one patient who underwent hepatic resection for intrahepatic CCA developed persistent hyperbilirubinemia, later diagnosed as bile leakage. None of the patients with mass-forming CCA developed persistent hyperbilirubinemia. Thirteen of the 14 cases (92.9%) who developed post-operative liver failure underwent right-sided hepatectomy. None of the patients who underwent minor hepatic resection developed persistent hyperbilirubinemia.

*Relationship between pattern of persistent hyperbilirubinemia and patients outcome*

The most common pattern of persistent hyperbilirubinemia was ‘Decreasing’. The respective number of an decreasing, increasing, ‘V’, peak, and persistent pattern was 9 (32.1%), 7 (25%), 7 (25%), 3 (10.7%), and 2 (7.1%). Nearly all of the patients (6/7, 85.7%) with the ‘V’ pattern were diagnosed with clinically significant post-operative liver failure, while a minority of those with a decreasing pattern (22.2%) developed liver failure (Table 2).

*Survival of the patients*

The median survival time for all the patients was 1,302 (1,159.3 1,444.7) days. The respective 1- and 3-year survival rate was 80 % (71.7-86.4%) and 55% (45.5 63.6%). In this cohort, none of the patients survived to 5 years after the operation. There were, however, differences in survival time with respect to the pattern of serum total bilirubin (Figure 3). The respective median survival time of the patients with normal bilirubin level, the ‘V’ pattern, and the other patterns was 1,589 (1,212.1-1,965.9), 469 (230.3- 707.7), and 792 (605.1- 978.9) days.

**Table 1:** Clinical and Operative Data

Variables	Normal (n= 88 )	Liver failure (n= 14 )	Bile leakage (n= 4 )	Stricture/ Cholangitis (n= 1 )	Unspecified (n= 9 )
Age (mean. SD)	62.8 (8.4)	58.4 (6.1)	65.0 (5.7)	68 (-)	58.4 (9.6)
Gender (male)(%)	50 (56.8)	10 (71.4)	4 (100)	1 (100)	7(77.8)
Location (%)					
Intrahepatic	51 (57.9)	0 (0)	1 (25)	0 (0)	0(0)
Perihilar	37 (42.0)	14 (100)	3 (75)	1 (100)	9(100)
Type of CCA (%)					
MF	12 (13.6)	0 (0)	0 (0)	0 (0)	0(0)
PI/ FN	18 (20.5)	9 (64.3)	1 (25)	0 (0)	5(55.6)
IG/ PP	58 (65.9)	5 (35.7)	3 (75)	1 (100.0)	4(44.4)
Procedure (%)					
Right hepatectomy	38 (43.2)	4 (28.6)	2 (50.0)	1 (100.0)	2(22.2)
Extended Right hepatectomy	9 (10.2)	5 (35.7)	1 (25.0)	0 (0.0)	3(33.3)
Right trisectionectomy	5 (5.7)	4 (28.6)	1 (25.0)	0 (0.0)	2(22.2)
Left hepatectomy	28 (31.8)	0 (0.0)	0 (0.0)	0 (0.0)	1(11.1)
Extended Left hepatectomy	2 (2.3)	0 (0.0)	0 (0.0)	0 (0.0)	1(11.1)
Left trisectionectomy	1 (1.1)	1 (7.1)	0 (0.0)	0 (0.0)	0(0.0)
Other	5 (5.7)	0 (0.0)	0 (0.0)	0 (0.0)	0(0.0)
Vascular resection (%)	3 (3.4)	3 (21.4)	0 (0.0)	0 (0.0)	2(22.2)
Vascular inflow occlusion (%)	35 (39.8)	4 (28.6)	3 (75)	1 (100)	3(33.3)
IVC Clampimg (%)	20 (22.7)	3 (21.4)	1 (25.0)	0 (0.0)	2(22.2)
EBL (ml)(SD)	524.2 (351.6)	880.0 (775.2)	675.0 (562.0)	1300.0 (-)	1088.9 (506.1)
LOS(days)(SD)	11.2 (5.1)	21.2 (9.8)	18.0 (9.0)	45 (-)	13.0 (5.0)
Preoperative laboratory investigation (median: min-max)					
Total biliarubin (mg/dl)	0.7 (0.2-3.5)	4.1 (1.2- 8.3)	1.65 (0.5-3.8)	19.3 (19.3 -19.3)	4.9 (1.8 -8.6)
AST (u/L)	196 (23 -1290)	469 (177-1283)	623 (139-2552)	1289 (1289-1289)	493 (114-738)
ALT (u/L)	125 (35-1190)	275.5 (92-633)	359 (62-1568)	746 (746-746)	302 (72-632)
ALP (u/L)	94.5 (39- 511)	146.5 (61-350)	132.5 ( 87-225)	110 (110- 110)	174 (109-605)
Albumin (g/dl)	2.8 (1.7- 4.3)	2.6(1.9-3.3)	2.9 (2.4- 4.4)	2 (2-2)	2.4 (2.2- 3.5)
Cholesterol (mg/dl)	135.5 (60-275)	119 (89-205)	136.5 (98-175)	90 (90-90)	113 (60-156)
Postoperative Total bilirubin					
Postoperative day 1	1.4 (0.3-5.1)	5.75 (2-16)	3.2 (1-6.6)	27.7 (27.7-27.7)	6.6 (3.1-12.9)
Postoperative day 3	1 (0.3-4.4)	6.45 (1.3-10.1)	3.45 (0.9-6.7)	24.8 (24.8-24.8)	6 (3-15.2)
Postoperative day 5	0.9 (0.2-2.7)	6.9 (2.9-12.6)	4.5 (3.3-7)	25.9(25.9-25.9)	5.5 (3.3-17.2)
Survival					
median (days)	1589 (1212-1965)	469 (230-707)	500 (193-806)		1302 (1123-1481)
1 years-survival (%)	0.81 ( 0.72- 0.88)	0.64 (0.34-0.83)	1		0.89 (0.43- 0.98)
3 years-survival (%)	0.62 (0.51- 0.71)	0.21 (0.05- 0.44)	.	.	0.56 (0.20- 0.80)

CCA, cholangiocarcinoma; MF, mass-forming; PI, periductal-infiltration; FN, flat and nodular infiltration; IG, intraductal- growth; PP, papillary-polypoid; IVC, inferior venacava; EBL, estimated blood loss; SD, standard deviation; LOS, length of hospital stays; AST, aspartate aminotransferase; ALT, Alanine aminotransferase; ALP, alkaline phosphatase

**Table 2:** Association between Patterns of Bilirubin Level and Causes of Hyperbilirubinemia

Cause	Pattern				
	Increasing (n= 7)	Decreasing (n=9)	Peak (n=3)	V-shape (n=7)	Persist (n=2)
Liver Failure	4 (57.1%)	2 (22.2%)	2 (66.7%)	6 (85.7%)	0 (0%)
Bile leakage	1 (14.3%)	1 (11.1%)	1 (33.3%)	1 (14.3%)	0 (0%)
Stricture/ cholangitis	0 (0%)	0 (0%)	0 (0%)	0 (0%)	1 (50%)
Unspecified	2 (28.6%)	6 (66.7%)	0 (0%)	0 (0%)	1 (50%)

## Discussion

The current study reported the causes of persistent hyperbilirubinemia in patients who underwent hepatectomy for cholangiocarcinoma (CCA). We proposed five patterns of persistent hyperbilirubinemia and found that the ‘V’ pattern was associated with post-operative liver failure and poor survival. Until now, there has been no study investigating the association between these patterns and patient outcome. 

The most serious complication after hepatectomy is post-operative liver failure (Dumitrascu et al., 2016; Nakamura et al., 2016). We found that the incidence of post-operative liver failure for CCA was 12%—all cases of which were perihilar CCA, probably because the perihilar type requires a more radical surgery leading to more liver volume loss. Most of the patients with a perihilar tumor have biliary obstruction at the time of diagnosis that subsequently causes some degree of liver dysfunction (Miyasaki et al., 2015). The finding is consistent with a previous report on the very high rate of post-operative liver failure after hepatectomy for perihilar CCA (Dumitrascu et al., 2016). Given the high rate of liver failure, high mortality, and preventability of this condition, finding an early predictor of this condition is warranted. 

Persistent hyperbilirubinemia is an early sign of liver failure (Kuramitsu et al., 2016; Liu et al., 2017); however, there are several conditions that can cause post-operative persistent hyperbilirubinemia, including bile leakage, biliary stricture, and acute cholangitis (Russel, 2015). We found that the most common cause of persistent hyperbilirubinemia after hepatic resection for CCA was liver failure compared to bile leakage and/or biliary stricture. This finding contradicts Dumitrascu et al.(2016) who reported the rate of post-operative bile leakage after surgery for perihilar CCA was more than 20% as high as of liver failure (Dumitrascu et al., 2016). The reasons for the different findings may be (i) the difference in the definition used for bile leakage, (ii) the nature of the disease, and (iii) the surgical technique. The high rate of post-operative liver failure among patients with persistent hyperbilirubinemia at our center suggests that all perihilar CCA patients with persistent hyperbilirubinemia should be monitored and treated as having post-operative liver failure until proven otherwise. Despite the intensive investigation, we found some patients with unspecified causes of persistent hyperbilirubinemia, which gradually improved over time. The condition may occur when the synthetic functions of the liver have recovered from the process of hepatocyte regeneration while regeneration of the bile canaliculi remains incomplete.

The serum bilirubin level indicates the status of the liver. The pattern of the bilirubin level can be used to predict post-operative complication. After hepatectomy, the bilirubin level usually rises for 1-2 days as a result of injuries to the intra-operative liver parenchymal, both direct injuries during parenchymal transection and indirect injuries from vascular inflow occlusion (Russel, 2015). The ischemic liver parenchyma left in the body continue, moreover, to produce liver enzymes and bilirubin: at POD3, this process usually resolves. We found that re-rising of the serum bilirubin level after POD3, resulting in a ‘V’ pattern, indicated a secondary event after the recovery phase—the most common event being postoperative liver failure. This pattern is thus predictive of a poor long-term outcome. An explanation for this finding is that the greater the degree of liver resection required, the more extensive the tumor, which is directly associated with poorer patient survival. This is the first study to explicitly identify the relationship between a pattern of persistent hyperbilirubinemia and poorer survival. We suggest that aggressive monitoring and investigation should be performed for the patient with the ‘V’ pattern in order to achieve early detection and treatment of post-operative liver failure. 

To the best of our knowledge, this is the first study—with a relatively large sample size—using the pattern of serum bilirubin for prediction of liver failure and long-term outcome of patients who underwent hepatectomy for cholangiocarcinoma. All patients were followed up long enough to determine the cause of the persistent hyperbilirubinemia. The study must, however, be interpreted carefully. Whereas most of the clinical and operative data were recorded prospectively, the review was performed retrospectively. During the study period, there was no uniform guideline regarding the future liver remnant, indication of portal vein embolization, and the standardization of the operation.

In conclusion, the present study provides a picture of persistent hyperbilirubinemia after hepatectomy for cholangiocarcinoma. The proportion of post-operative liver failure was 12 percent. The pattern of serum bilirubin level could be used as a predictor of liver failure and long-term patient outcome. The ‘V’ pattern was significantly associated with a high rate of liver failure and poor survival. 
